# Reformulation of Public Help Index* θ* Using Null Player Free Winning Coalitions

**DOI:** 10.1007/s10726-021-09769-4

**Published:** 2021-11-21

**Authors:** Izabella Stach

**Affiliations:** AGH University of Science and Technology, Al. Mickiewicza 30, 30-059 Krakow, Poland

**Keywords:** Power indices, Public goods, Simple game, Weighted game

## Abstract

This paper proposes a new representation for the Public Help Index *θ* (briefly, PHI *θ*). Based on winning coalitions, the PHI *θ* index was introduced by Bertini et al. in (2008). The goal of this article is to reformulate the PHI *θ* index using null player free winning coalitions. The set of these coalitions unequivocally defines a simple game. Expressing the PHI *θ* index by the winning coalitions that do not contain null players allows us in a transparent way to show the parts of the power assigned to null and non-null players in a simple game. Moreover, this new representation may imply a reduction of computational cost (in the sense of space complexity) in algorithms to compute the PHI *θ* index if at least one of the players is a null player. We also discuss some relationships among the Holler index, the PHI *θ* index, and the *g*^*np*^ index (based on null player free winning coalitions) proposed by Álvarez-Mozos et al. in (2015).

## Introduction

In the literature, a power index is defined as a measure that is meant to assess the a priori power, as the influence, or as the payoff expectation of players in a simple game.

This paper concentrates on the Public Help Index *θ* (briefly, PHI *c* or just *θ*) introduced by Bertini et al. (2008) and based on winning coalitions. The PHI *θ* index was born as a modification of the Public Good Index (briefly, PGI) based on minimal winning coalitions. The PGI index was introduced by Holler (1982); for this reason, it is also known as the Holler index. Even though the structure of the two indices mentioned above are very similar, their characterizations differ (see Sect. 5, for example, where a comparison of two indices can be found). The PHI *θ* index takes part of the so-called public power indices (see Bertini and Stach (2015) and Stach (2016) as *θ* is well-defined in the social context where goods are public. The PHI *θ* index considers all winning coalitions, not only minimal winning coalitions (as in the PGI index). Hence, as Bertini and Stach (2015) remarked, “*θ* rather describes power relationships in the consumption of public goods, whereas the PGI analyzes the production of public goods. In production, one must take care to exclude free-riding; this is why the PGI considers minimal winning coalitions; in the consumption of public goods, you cannot avoid free-riding.”

The PHI *θ* index is much closer to the König and Bräuninger (1998) index (briefly, *KB*) or the *Z* index introduced by Nevison (1979). Both indices (*KB* and Z) are not only based on winning coalitions and closely related to the Banzhaf (1965) index but are also proportional to each other and to the *θ* index. In particular, the quotient of each of these indices and the sum of its values taken over of all players is equal to the *θ* index, which is not difficult to show (see Bertini et al. (2013) and Stach (2016), for example). Moreover, if we consider voting rules to make yes/no choices (acceptance and rejection) by a voting body and assume that all vote configurations are equally probable, then the *KB* index for particular voter *i* is interpreted as the conditional probability that “*i* is successful” given “the proposal is accepted” (see Valenciano et al. (2004), for example). The *Z* index was defined by Nevison (1979) as an absolute measure of satisfaction. So, from this point of view, the PHI *θ* index can be seen as the relative measure of being successful (having the result for which one voted).

PHI *θ* takes all winning coalitions into account. This also provides a non-negative power to null players (if any), reducing the power of others. However, considering the consumption of public goods and social help, we cannot exclude free-riders in many cases (especially when we consider the health of the whole society, an important issue nowadays during the COVID-19 pandemic, for example). Conventional methods that measure the voting power of players, such as the Banzhaf (1965), Shapley and Shubik (1954) indices, for example, are unsuitable for this purpose. When distributing the public good, they do not take the null players into account. The *θ* index is solidary with weak players (not only null players), while the mentioned above indices are not.

In their paper that introduced the solidarity value, Nowak and Radzik (1994) provided a very convincing example about solidarity with “weaker” players. In this example, there are three brothers who live together. One of them is a disabled person who can contribute nothing to any coalition. The Shapley-Shubik, Banzhaf, and PGI indices assign zero power to the “weak” brother. Nowak and Radzik (1994) posed the following question: “Should the disabled brother leave his family?” The *θ* index represents a solidary with the “weak” brother (see Example 5 in Sect. 4).

The object of this article is to find a new formula for PHI *θ* based on so-called null player free winning coalitions, a concept first time used in Álvarez-Mozos et al. (2015). In this way, we can show the parts of the power assigned to the null and non-null players in the presence of null players in a simple game in a transparent way. It should be noted that the set of null player free winning coalitions unequivocally defines a simple game. We also discuss some relationships among the Holler index, the *θ* index, and the *g*^*np*^ index (based on null player free winning coalitions) proposed by Álvarez-Mozos et al. (2015). In particular, we prove that just like the PHI *θ* index also the *g*^*np*^ index satisfies the dominance and bicameral meet properties. In addition, the *θ* and *g*^*np*^ indices are equal when a game is free of null players (see Sect. 2.1).

In the forthcoming paper by Stach and Bertini (2021), a representation that is based on the information contained in a set of null player free winning coalitions is given for some well-known indices like the Banzhaf (1965) index, the Rae (1969) index, Coleman’s (1971) indices to prevent action and to initiate action, Nevison’s (1979) *Z* index, and the König and Bräuninger (1998) index. Moreover, some relationships among the Banzhaf index and these power indices are also established by using the new reformulations.

The rest of the paper is structured as follows. In Sect. 2, we provide some basic definitions and notations. Section 3 contains the new formula of PHI *θ* using the concept of null player free winning coalitions. In Sect. 4, some examples of a simple game are considered to compare PHI *θ* with the *g*^*np*^ and PGI indices in order to reveal the possible application fields in which the *θ* index is suitable and to show how the new formula of PHI *θ* can be useful in its algorithmic calculation in games with at least one null player and in games that are determined by a set of null player free winning coalitions. Section 5 is dedicated to comparing the three power indices (*h*, PHI *θ*, and *g*^*np*^) by taking some desirable properties of power indices into account. With Sect. 6, we conclude.

## Definitions and Notations

A *cooperative n-person game* is a pair (*N*, *v*) where $N = \{ 1,2,...,n\}$ is a finite set of *n* players and *v* is real-valued function $v:2^{N} \to R$ with $v(\emptyset ) = 0$. $2^{N}$ denotes the set of all subsets of *N*. Each $S \in 2^{N}$ is called a *coalition* and *N* is called the *grand coalition*. Hereafter we call both (*N*, *v*) and *v* a game since *N* is inherent in the definition of *v*.$v(S)$ stands for the *worth* of coalition *S* in game *v*. |*S*|= *s* denotes the cardinality of *S*, so |*N*|= *n*. A cooperative game is *monotonic* if $v(S) \le v(T)$ is true whenever $S \subset T \subseteq N$.

A *simple* game is a monotonic^1^ cooperative game such that $v(S) \in \{ 0,1\}$ for all $S \subseteq N$ and $v(N) = 1$. In simple games, we call coalitions with property $v(S) = 1$ *winning coalitions*, while those with $v(S) = 0$ are called *losing coalitions*. By *W*, we denote the set of all winning coalitions in simple game (*N*, *v*). Any simple game may be unequivocally described by its set of winning coalitions. *W*_*i*_ stands for the set of all winning coalitions that contain player *i*. A simple game is *proper* if the following condition holds: $\forall S \subseteq N$ if $v(S) = 1$, then $v(N\backslash S) = 0$. In this paper, we analyze only proper simple games (for a proper simple game, see (Stach 2011), for example). By *S*_*N*_, we denote the set of all simple games on *N*.

A player $i \in N$ is *critical* in $S \in W$ if $(S\backslash \{ i\} ) \notin W$. Coalition $S \in 2^{N}$ is called a *minimal winning coalition* if all $i \in S$ are critical. By *W*^*m*^*,* we denote the set of all minimal winning coalitions, and $W_{i}^{m}$ stands for the set of all minimal winning coalitions that contain player *i.*

A player $i \in N$ is called a *null player* if, for all coalitions, $S \in 2^{{N\backslash \{ i\} }}$ $v(S \cup \{ i\} ) - v(S) = 0$. The amount $v(S \cup \{ i\} ) - v(S)$ is called a *marginal contribution* of player *i* to $S \in 2^{{N\backslash \{ i\} }}$. A coalition $S \in 2^{N}$ is called a *null player free winning coalition* if $S \in W$ and none of its members is a null player. By $W^{n - }$, we denote the set of all null player free winning coalitions, and $W_{i}^{n - }$ denotes the set of all null player free winning coalitions that contain player *i*. Like *W* and *W*^*m*^, $W^{n - }$ determines an unequivocally simple game.

A simple game (*N*, *v*) is called a *weighted game* and is represented by $[q;\,w_{1} ,\,...,\,w_{n} ]$ if there exists a non-negative vector of the weights of players $(w_{1} ,\,...,\,w_{n} )$ and a majority quota *q* $\sum\limits_{i \in N} {w_{i} } \ge q > 0$ such that $v(S) = 1$ if and only if $\sum\limits_{i \in S} {w_{i} } \ge q$. A weighted game $[q;\,w_{1} ,\,...,\,w_{n} ]$ is proper if $q > \frac{1}{2}\sum\limits_{i \in N} {w_{i} }$.

A *power index f* is a function that assigns a unique vector $f(v) = (f_{1} (v),f_{2} (v),\,...\,,f_{n} (v))$ to each simple game $v \in S_{N}$. Each component $f_{i} (v)$ is a measure of the (a priori) player *i*’s power in the game *v* — specified by its player set *N* and its characteristic function *v*. So, the power index is an attempt to quantify the differences in power between players. The bigger the value $f_{i} (v)$, the more significant is the power of player *i* in simple game *v*.

### Some considerated power indices

In this section we give the definitions of the three power indices considered in this paper: the *θ* index, the *g*^*np*^ index, and the Public Good Index (*h*).

Let $v \in S_{N}$ and $i \in N$. Public Help Index *θ* (introduced and axiomatized by Bertini, et al. (2008)) is given by:

$$\theta_{i} (v) = \frac{{|W_{i} |}}{{\sum\nolimits_{j \in N} {|W_{j} |} }}.$$

The *g*^*np*^ power index (also called null player free index) was defined in (Álvarez-Mozos et al. 2015) as follows. Given simple game $v \in S_{N}$,

$$ g_{i}^{np} (v) = \frac{{|W_{i}^{n - } |}}{{\sum\limits_{j \in N} {|W_{j}^{n - } |} }}{\text{ for every }}i \in N. $$

For an axiomatic characterization of this index, see (Álvarez-Mozos et al. 2015).

The Public Good Index (also called Holler index), *h*, was proposed by Holler (1982). For all $v \in S_{N}$ and $i \in N$ the *h* index is defined as follows:

$$h_{i} (v) = \frac{{|W_{i}^{m} |}}{{\sum\limits_{j \in N} {|W_{j}^{m} |} }}$$.

For an axiomatic characterization of the Public Good Index, see Holler and Packel (1983). Moreover, Napel in (1999, 2001) showed the independence and non-redundancy of the Holler and Packel axioms.

### Some Proporties of Power Indices in Simple Games

In this section we provide definitions of some well-known and desirable properties of power indices in simple games. In Sect. 5, we will compare the power indices defined in Sect. 2.1 (*θ*, *g*^*np*^, and *h*) by taking these properties into consideration.

We say that *f* satisfies the following:

- the *efficiency property* if, for all (*N*, *v*), $\sum\limits_{i \in N} {f_{i} (v)} = 1$;
- the *null player property* if $f_{i} (v) = 0$ for all (*N*, *v*) and each null player $i \in N$;
- the *null player removable property* if, for all simple games $v^{\prime}$ arising from *v* by eliminating the null players, $f_{i} (v^{\prime}) = f_{i} (v)$ holds for each non-null player $i \in N$;
- the *symmetry* (*anonymity*) *property* if, for all simple games (*N*, *v*), each player $i \in N$, and every permutation $\pi :N \to N$, the following equation holds: $f_{i} (v) = f_{\pi (i)} (\pi (v))$, where a permuted game is defined as $(\pi (v))(S) = v(\pi^{ - 1} (S))$;
- the ***non-negativity property* if** $f_{i} (v) \ge 0$ for all (*N*, *v*) and $i \in N$;
- the *dominance (local monotonicity) property* if, for all weighted games $[q;\,w_{1} ,\,...,\,w_{n} ]$ and any two distinct players $i,j \in N$, condition $w_{i} \ge w_{j}$ implies $f_{i} (v) \ge f_{j} (v)$;
- the *donation property* if, for two weighted games *v* = $[q;w_{1} ,...,w_{i} ,...,w_{j} ,...,w_{n} ]$ and *v*’ = $[q;w_{1} ,...,w_{i} - \delta ,...,w_{j} + \delta ,...,w_{n} ]$ where $0 < \delta \le w_{i}$ and $i \ne j$, $f_{i} (v) \ge f_{i} (v^{\prime})$ holds;
- the *redistribution property* if, for two weighted games *v* = $[q;w_{1} ,...,w_{n} ]$ and *v*’ = $[q;w^{\prime}_{1} ,...,w^{\prime}_{n} ]$ where *v’* arising from *v* by the redistribution of weights ( $\sum\limits_{i = 1}^{n} {w_{i} } = \sum\limits_{i = 1}^{n} {w^{\prime}_{i} }$), then player *i* who decreases his weight ( $w_{i} > w^{\prime}_{i}$) does not increase his power $f_{i} (v) \ge f_{i} (v^{\prime})$;
- the *fattening property* if, for two weighted games *v* = $[q;w_{1} ,...,w_{i} ,...,w_{n} ]$ and *v*’ = $[q;w_{1} ,...,w_{i} + \delta ,...,w_{n} ]$ where $i \in N$ and $\delta > 0$, $f_{i} (v) \le f_{i} (v^{\prime})$ holds;
- the *strong monotonicity* property if, for two simple games *v* and *v*’ having the same grand coalition *N* and if *i* is a player such that $v(S) - v(S\backslash \{ i\} ) \ge v^{\prime}(S) - v^{\prime}(S\backslash \{ i\} )$ for each $S \in 2^{N}$, then $f_{i} (v) \ge f_{i} (v^{\prime})$ holds;
- the *bicameral meet property* if, for three simple games $$(N_{1} ,v_{1} )$$, $$(N_{2} ,v_{2} )$$, $$(N,v)$$ such that $$N_{1} \cap N_{2} = \emptyset$$, $$N = N_{1} \cup N_{2}$$ and $$W(v)$$$$= \{ S \in 2^{N} :S \cap N_{1} \in W(v_{1} )$$ and $$S \cap N_{2} \in W(v_{2} )\}$$, $$\frac{{f_{i} (v_{1} )}}{{f_{j} (v_{1} )}} = \frac{{f_{i} (v)}}{{f_{j} (v)}}$$ holds for any two non-null players $$i,j \in N_{1}$$;
- the *transfer property* if, for all $v_{1} ,\,v_{2} \in S_{N}$ and each player $i \in N$, the following equation $f_{i} (v_{1} \wedge v_{2} ) + f_{i} (v_{1} \vee v_{2} ) = f_{i} (v_{1} ) + f_{i} (v_{2} )$ holds, where two simple games $(v_{1} \wedge v_{2} )$ and $(v_{1} \vee v_{2} )$ are defined as follows:
  $$ W(v_{1} \wedge v_{2} ) = \{ S \in 2^{N} :S \in W(v_{1} )\,{\text{ and }}S \in W(v_{2} )\} , $$
  $$ W(v_{1} \vee v_{2} ) = \{ S \in 2^{N} :S \in W(v_{1} )\,{\text{ or }}\,S \in W(v_{2} )\} . $$

## A New Formula for the Public Help Index

Let us consider a simple game $v \in S_{N}$ such that $n \ge 2$. Let *k* stand for the number of null players in *v*, $0 \le k < n$. By *N*^*n‒*^, we denote the set of non-null players in *v*, it means the set without nulls. By the way, the coalition *N*^*n‒*^ is a carrier in the sense of Shapley (1953).

### Theorem 1.

If $v \in S_{N}$ and $0 \le k < n$, then the PHI *θ* index can be expressed as follows:

1 $$ \theta_{i} (v) = \left\{ {\begin{array}{*{20}l} {\frac{{|W_{i}^{n - } |}}{{\sum\limits_{j \in N} {|W_{j}^{n - } | + \frac{k}{2}|W^{n - } |} }}} \hfill & {{\text{if }}i \in N^{n - } } \hfill \\ {\frac{{|W^{n - } |}}{{2\sum\limits_{j \in N} {|W_{j}^{n - } | + k|W^{n - } |} }}} \hfill & {{\text{if }}i{\text{ is a null player}}.} \hfill \\ \end{array} } \right. $$

### *Proof.*

Let us fix an *n*-player simple game with *k* null players ($0 \le k < n$) such that $W \ne \emptyset$. The set of all winning coalitions (*W*) can be seen as the union of two disjoint subsets: $W = W^{n - } \cup (W\backslash W^{n - } )$ where $W\backslash W^{n - }$ is the set of all winning coalitions that contain at least one null player. Note that, if $0 < k < n$ holds in a simple game, then $N \in (W\backslash W^{n - } )$.

All winning coalitions with null players $(W\backslash W^{n - } )$ can be simply obtained from null player free winning coalition $W^{n - }$. Namely, if we have *k* null players, we can obtain $(2^{k} - 1)$ different non-empty coalitions that contain only null players. Null players without other players cannot form a winning coalition. A null player (or a coalition of null players) can take part in winning coalitions if he/she joins one of the null player free coalitions. So, if we have $|W^{n - } |$ null player free coalitions in a game, it is possible to create $|W^{n - } |(2^{k} - 1)$ different winning coalitions with at least one null player. So, the number of all winning coalitions in a simple game is given by the following equation: $|W| = |W^{n - } | + |W^{n - } |(2^{k} - 1)$. From this, we immediately obtain: $|W| = 2^{k} |W^{n - } |$.

Let us assume that player *i* is a null player in game *W*. Thus, in total, *i* takes part in $2^{k - 1}$ different coalitions with all null players, and *i* belongs to $|W^{n - } |2^{k - 1}$ winning coalitions. Hence, $|W_{i} | = |W^{n - } |2^{k - 1}$ and *k* null players belong to $\sum\limits_{{j \in N,j{\text{ is a null player}}}} {|W_{j} |}$ =  $k|W^{n - } |2^{k - 1}$ winning coalitions in total.

Now, let us assume that *i* is a non-null player in game *W*, $i \in N^{n - }$. So, in total, *i* takes part in $|W_{i}^{n - } |$ null player free coalitions, and *i* belongs to $|W_{i}^{n - } |(2^{k} - 1)$ winning coalitions with at least one null player. Hence, $|W_{i} | = |W_{i}^{n - } | + |W_{i}^{n - } |(2^{k} - 1) = |W_{i}^{n - } |2^{k}$. If we take all non-null players into consideration, we have $\sum\limits_{{j \in N^{n - } }} {|W_{j} |}$ =  $\sum\limits_{{j \in N^{n - } }} {|W_{j}^{n - } |2^{k} }$ =  $2^{k} \sum\limits_{{j \in N^{n - } }} {|W_{j}^{n - } |}$.

From the above considerations for null and non-null players, we have $$\sum\limits_{j \in N} {|W_{j} |} =$$$$\sum\nolimits_{{j \in N^{n - } }} {|W_{j} |} + \sum\limits_{{j \in N,j{\text{ is a null player}}}} {|W_{j} |}$$  = $$2^{k} \sum\nolimits_{{j \in N^{n - } }} {|W_{j}^{n - } |}$$  + $$k|W^{n - } |2^{k - 1}$$. Now, we can express the PHI *θ* index for all $$v \in S_{N}$$ as follows:

$$ \theta_{i} (v) = \left\{ {\begin{array}{*{20}l} {\frac{{2^{k} |W_{i}^{n - } |}}{{2^{k} \sum\limits_{j \in N} {|W_{j}^{n - } | + k2^{k - 1} |W^{n - } |} }}} \hfill & {{\text{if }}i \in N^{n - } } \hfill \\ {\frac{{2^{k - 1} |W^{n - } |}}{{2^{k} \sum\limits_{j \in N} {|W_{j}^{n - } | + k2^{k - 1} |W^{n - } |} }}} \hfill & {{\text{if }}i{\text{ is a null player}}{.}} \hfill \\ \end{array} } \right. $$

We can still simplify the above formula by dividing the numerator and denominator of the first part (if $i \in N^{n - }$) by $2^{k}$ and then similarly dividing the numerator and denominator of the second part (if $i \notin N^{n - }$) by $2^{k - 1}$. After these operations, Formula (1) easily follows. □

Note that, if *k* = 0 in a simple game (i.e., all players are non-null players), the above Formula (1) of the PHI *θ* index is equal to the *g*^*np*^ index proposed by Álvarez-Mozos (2015) (see also Sect. 2).

Thanks to (1), for a given simple game (*N*, *v*) with *k* null players, we can easily separate the part of the power assigned by PHI *θ* to all null players from the part assigned to non-null players (see Corollary 1).

### Corollary 1

If $v \in S_{N}$ and $0 \le k < n$, then the total power assigned to all null players (*TPNP*) is equal to.

2 $$ TPNP = \frac{k|W|}{{2\sum\limits_{j \in N} {|W_{j} |} }} = \frac{{k|W^{n - } |}}{{2\sum\limits_{j \in N} {|W_{j}^{n - } | + k|W^{n - } |} }}. $$

If $0 < k < n$, then this value is equal to

3 $$ TPNP = \frac{1}{{\frac{2}{k}\sum\limits_{j \in N} {\frac{{|W_{j}^{n - } |}}{{|W^{n - } |}} + 1} }}. $$

### *Proof.*

The demonstration is immediate from Formula (1) (see Theorem 1 and its proof). Note that for each null player *i* we have $|W_{i} | = 2^{k - 1} |W^{n - } | = \frac{|W|}{2}$ (see the proof of Theorem 1) which also results from the identity proposed by Dubey and Shapley (1979, p. 127).

As a consequence of Corollary 1, we can also immediately calculate the total power assigned by the PHI *θ* index to all non-null players (see Corollary 2).

### Corollary 2

If $v \in S_{N}$ and $0 \le k < n$, then the total power assigned to all non-null players (*TPNNP*) is equal to.

$$\frac{{\sum\limits_{j \in N} {|W_{j}^{n - } |} }}{{\sum\limits_{j \in N} {|W_{j}^{n - } | + \frac{k}{2}|W^{n - } |} }}$$.

### *Proof.*

The demonstration immediately follows from Formula (1) by summing over $\theta_{j} (v)$ for all $j \in N^{n - }$. Since the denominator is the same for all $j \in N^{n - }$, one just needs to sum over $|W_{j}^{n - } |$ in the numerator. □

## Public Help Index and Null Player Free Index in Examples

In this section, we present some examples to illustrate the use of new Formula (1) to compare PHI *θ* and *g*^*np*^ as well as to show the application fields in which the *θ* index is suitable.

It is worth noticing that, in all of the examples presented below as well as in the general case of simple games with null players, the PHI *θ* can be calculated from both the original formula and the new one. However, when we would like to have an algorithm to calculate the PHI *θ* index in an automatic way, then Formula (1) seems more suitable for games with the presence of null players. This formula explicitly induces to identify null players first, eliminate them from consideration, and find the set of winning coalitions without them (which means the set of null player free winning coalitions), thus saving the space complexity of an algorithm. This is particularly valid for simple games with a large number of players and null players.

Furthermore, when a simple game is defined by the set of null player free winning coalitions, the calculation of the *θ* index by Formula (1) is immediate.

### ***Example 1***

Let us consider weighted game [3; 3, 1, 0]. In this game, we have three players. The game can model the national health service (i.e., a publicly funded healthcare system) of a certain country. Player 1 (with a weight equal to $w_{1} = 3$) can represent those groups of the society that guarantees themselves health service by paying a regular fee. Player 2 (with $w_{2} = 1$) can represent a group that contributes to the health service, but this is insufficient to guarantee all services. Player 3 ($w_{3} = 0$) represents a group that is unable to guarantee themselves health services (the homeless, underprivileged, migrants, and unemployed, for example).

In this game, Players 2 and 3 are null players. Their marginal contribution to all winning coalitions is null. During the time of a pandemic, we cannot exclude null players for the common prosperity in the “division” of the rights to the health service (this means players that do not contribute anything). According to Public Help Index *θ* and Formulas (2) and (3), the total power assigned to the null players is equal to.

$$TPNP = \frac{1}{{\frac{2}{k}\sum\limits_{j \in N} {\frac{{|W_{j}^{n - } |}}{{|W^{n - } |}} + 1} }} = \frac{1}{{\frac{2}{2}(\frac{1}{1} + \frac{0}{1} + \frac{0}{1}) + 1}} = \frac{1}{2}$$.

So, since all non-null players are symmetric, due to anonymity property, each of the null player’s power is equal to $\theta_{2} (v) = \theta_{3} (v) = \frac{1}{4}$. As the *θ* index satisfies the efficiency property (see Sect. 2), the power of Player 1 immediately follows $\theta_{1} (v) = 1 - \frac{1}{4} - \frac{1}{4} = \frac{1}{2}$; this is in line with the calculation made using Formula (1).

Table 1 shows the distribution of power according to the three indices considered in this paper.

**Table 1:** Power index distributions in Example 1

Power index	Player		
	1	2	3
Public Help Index *θ*	1/2	1/4	1/4
*g*^*np*^	1	0	0
Public Good Index *h*	1	0	0

### ***Example 2***

Let us consider another example of a weighted game that illustrates how much faster we can find the distribution of the PHI *θ* index in simple games with a presence of null players. Namely, let us consider the weighted game [9; 5, 5, 1, 1, 1].

In this game, we have three null players: 3, 4, and 5. So, *k* = 3. Next, we have eight winning coalitions *W* = {{1, 2}, {1, 2, 3}, {1, 2, 4}, {1, 2, 5}, {1, 2, 3, 4}, {1, 2, 3, 5}, {1, 2, 4, 5}, {1, 2, 3, 4, 5}} and only one null player free winning coalition: {1, 2}, |*W*^*n‒*^|= 1. Thus, $|W_{i}^{n - } | = |W_{2}^{n - } | = 1$, $\sum\limits_{j \in N} {|W_{j}^{n - } |} = 2$. So, the calculation of the *θ* index by Formula (1) is immediate. $\theta_{1} (v) = \theta_{2} (v) = \frac{2}{7}$, $\theta_{3} (v) = \theta_{4} (v) = \theta_{5} (v) = \frac{1}{7}$.

This example, even with only five players, perfectly shows how the computation of PHI *θ* in games with numerous players can be simplified by applying the new formula. This means considering only null player free winning coalitions instead of all winning coalitions in the calculation of the *θ* index.

For example, in a weighted game, it is not that difficult to identify all null players (see Chakravarty et al. (2014, p. 234), for example). Namely, let a weighted game be given $[q;\;w_{1} ,w_{2} ,...,w_{n} ]$ where $w_{1} \ge w_{2} \ge \cdots \ge w_{n}$. Let $\alpha_{i} = \max \{ w(S):\;S \subseteq \{ 1,2,...,i\} {\text{ and }}w(S) < q\}$ be the maximum weight of any losing coalitions that is a subset of {1, 2, …, *i*} and $\beta_{i} = \sum\limits_{j = i}^{n} {w_{j} }$ denote the sum of all weights from player *i* to player *n*. According to the necessary and sufficient condition provided by Matsui and Matsui (2000), a player $i > 1$ is a null player if and only if $\alpha_{i - 1} + \beta_{i} < q$.

Concluding, the new formula of *θ* can reduce the space complexity of calculating the *θ* in simple games with at least one null player. Generally speaking, the space complexity of an algorithm (computer program) quantifies the amount of memory space that is required by an algorithm to run as a function of the length of the input (see Kuo and Zuo (2003), for example). Hence, a lower number of players in the input implies a reduction in the space complexity of an algorithm to calculate the PHI *θ* index.

### ***Example 3***

Let us consider a real-world political example of a voting system. Namely, the 1958 European Union voting system which is described by the weighted game [12; 4, 4, 4, 2, 2, 1]. In this system the approval of a decision required at least 12 votes of the total 17. The corresponding weights were four votes each for Germany, France and Italy, two votes each for the Netherlands and Belgium, and only one vote for Luxembourg.

The assessment of Luxembourg’s voting power is null by the standard power indices as those proposed by Shapley and Shubik (1953) and Banzhaf (1965). The role of Luxembourg in the Council of Ministers in the first period of the European Economic Community certainly was not null (see Mayer (2018), for example). Also, the Holler and *g*^*np*^ indices assign zero power to Luxembourg. Of course, the evaluation of voting power is sensitive to measurement concepts applied. The PHI *θ* index assigns the lowest non-null voting power to Luxembourg; i.e., 1/9 (see Table 2).

**Table 2:** Power index distributions in 1958 EU voting system (Example 3)

Power index	Players		
	Germany, France, Italy	Netherlands, Belgium	Luxembourg
Shapley-Shubik	14/60	9/60	0
Banzhaf	10/42	6/42	0
Public Good Index *h*	1/5	1/5	0
*g*^*np*^	6/28	5/28	0
Public Help Index *θ*	12/63	10/63	7/63

### ***Example 4***

Let us turn to the COVID-19 example and modify it a bit considering only two groups of players: Polish citizens, and the set of null players. The set of null players can represent groups of immigrants, unemployment, and closed businesses by COVID-19, for example. Each Polish citizen has weight equal to 1, and each null player has weight equal to 0. As was mentioned before in this kind of problem all players should have rights to have a medical service, access to the vaccine (where available), etc., and the surplus would be shared among the non-null players (subsidies for closed businesses, unemployment caused by COVID-19, etc.).

If so, we can apply the PHI *θ* index to share the total pandemic budget. Let us assume that in this weighted game, we have *m* Polish citizens ($0 < m < n$ and *m* is on the order of 37–38 millions) and *k* null players ($0 < k < m$).

Regardless of the adopted majority threshold, power indices such as Banzhaf, Shapley-Shubik, and *g*^*np*^, distribute the available budget equally among Polish citizens only. So each player gets 1/*m* of total medical service budget available.

The *θ* index distribution depends on the adopted majority quota and the number of null players. If we assume the absolute majority of the Polish citizens, then the game representation depends on whether *m* is an even or odd number. So, let us assume (without a loss of generality) that *m* is odd and the quota is placed at *q* = (*m* + 1)/2. Then, the game can be represented in the following way:

$$v = \left[ {\frac{m + 1}{2};\underbrace {1,\,1,\,...,\,1}_{m},\,\underbrace {0,\,0,\,...,\,0}_{k}} \right]$$, where $$n = m + k$$, $$0 < k < m$$, $$m > 0$$.

Note that the computations of the PHI *θ* index for each player in this game are easy using both, the new and former formulas. We use Eq. (1) here. So, let us find $|W^{n - } |$ and $|W_{i}^{n - } |$ for each non-null player *i* (Polish citizen). In this example, all null player free winning coalitions must have at least $\frac{m + 1}{2}$ non-null players. So, $$|W^{n - } | = \left( {\begin{array}{*{20}c} m \\ {{{(m + 1)} \mathord{\left/ {\vphantom {{(m + 1)} 2}} \right. \kern-\nulldelimiterspace} 2}} \\ \end{array} } \right) + \left( {\begin{array}{*{20}c} m \\ {{{(m + 1)} \mathord{\left/ {\vphantom {{(m + 1)} 2}} \right. \kern-\nulldelimiterspace} 2} + 1} \\ \end{array} } \right) + \cdots + \left( {\begin{array}{*{20}c} m \\ m \\ \end{array} } \right) = 2^{m - 1}$$ if *m* is an odd number. For each non-null player *i*, $$|W_{i}^{n - } | = \left( {\begin{array}{*{20}c} {m - 1} \\ {{{(m - 1)} \mathord{\left/ {\vphantom {{(m - 1)} 2}} \right. \kern-\nulldelimiterspace} 2}} \\ \end{array} } \right) + \left( {\begin{array}{*{20}c} {m - 1} \\ {{{(m - 1)} \mathord{\left/ {\vphantom {{(m - 1)} 2}} \right. \kern-\nulldelimiterspace} 2} + 1} \\ \end{array} } \right) + \cdots + \left( {\begin{array}{*{20}c} {m - 1} \\ {m - 1} \\ \end{array} } \right) = 2^{m - 2} + \frac{1}{2}\left( {\begin{array}{*{20}c} {m - 1} \\ {{{(m - 1)} \mathord{\left/ {\vphantom {{(m - 1)} 2}} \right. \kern-\nulldelimiterspace} 2}} \\ \end{array} } \right)$$ when *m* is an odd number. Therefore, using Formula (1), we obtain the following:

$$ \theta_{i} (v) = \left\{ {\begin{array}{*{20}c} {\frac{{1 + \frac{1}{{2^{m - 1} }}\left( {\begin{array}{*{20}c} {m - 1} \\ {{{(m - 1)} \mathord{\left/ {\vphantom {{(m - 1)} 2}} \right. \kern-\nulldelimiterspace} 2}} \\ \end{array} } \right)}}{{m\left( {1 + \frac{1}{{2^{m - 1} }}\left( {\begin{array}{*{20}c} {m - 1} \\ {{{(m - 1)} \mathord{\left/ {\vphantom {{(m - 1)} 2}} \right. \kern-\nulldelimiterspace} 2}} \\ \end{array} } \right)} \right) + k}}} & {{\text{if }}i \in N^{n - } {\text{ and }}m{\text{ is an odd number}}} \\ {\frac{1}{{m\left( {1 + \frac{1}{{2^{m - 1} }}\left( {\begin{array}{*{20}c} {m - 1} \\ {{{(m - 1)} \mathord{\left/ {\vphantom {{(m - 1)} 2}} \right. \kern-\nulldelimiterspace} 2}} \\ \end{array} } \right)} \right) + k}}} & {{\text{if }}i \notin N^{n - } {\text{ and }}m{\text{ is an odd number}}{.}} \\ \end{array} } \right. $$

As $$0 < \frac{1}{{2^{m - 1} }}\left( {\begin{array}{*{20}c} {m - 1} \\ {{{(m - 1)} \mathord{\left/ {\vphantom {{(m - 1)} 2}} \right. \kern-\nulldelimiterspace} 2}} \\ \end{array} } \right) < 1$$, then $\theta_{i} (v)$ results a higher solidary value here than in the case discussed in Example 1 (where the null player obtains half of what the non-null player does).

Then, if *m* tends towards infinity, then $$\frac{1}{{2^{m - 1} }}\left( {\begin{array}{*{20}c} {m - 1} \\ {{{(m - 1)} \mathord{\left/ {\vphantom {{(m - 1)} 2}} \right. \kern-\nulldelimiterspace} 2}} \\ \end{array} } \right)$$ tends towards zero, and the value assigned to each “weaker” player tends towards the value assigned to a non-null player. Of course, the same is valid when *m* is an even number.

### ***Example 5***

(“Three Brothers”). Players 1, 2, and 3 are brothers who live together. Players 1 and 2 can make a profit of one unit together (that is, *v*({1, 2}) = 1). Player 3 is a disabled person and can contribute nothing to any coalition. The *v*({*i*}) = 0 for each player *i* = 1, 2, 3. Therefore, *v*({1, 2, 3}) = 1 and *v*({1, 3}) = *v(*{2, 3}) = 0.

In this example, we have: $|W^{n - } |$= 1, $|W_{i}^{n - } |$= 1 for *i* = 1, 2. Player 3 is a null player. From Formula (1) or simply from the former one, we immediately obtain $\theta_{1} (v) = \theta_{2} (v) = \frac{2}{5}$, $\theta_{3} (v) = \frac{1}{5}$.

“Should the disabled brother leave his family?” This was a question that was posed by Nowak and Radzik (1994). If Players 1 and 2 take responsibility for their “weaker” brother (Player 3), then the PHI *θ*(*v*) index distribution (2/5, 2/5, 1/5) seems to be a “better” solution in this case than that which is given by the *g*^*np*^, Banzhaf, or Shapley-Shubik indices: (1/2, 1/2, 0).

## Public Help, Null Player Free, and Holler Indices in Comparison

The PHI *θ* and *g*^*np*^ indices were born as modifications of the Holler index (*h*). In this section, we compare the *θ*, *g*^*np*^, and *h* indices by taking the ranges of the indices and some desirable properties of the power indices into account. All of these indices have the same structure, but they based on different families of coalitions: winning coalitions (*W*), null player free winning coalitions (*W*^*n−*^), and minimal winning coalitions (*W*^*m*^), respectively. Given a player set *N*, each of these sets unequivocally determines the simple game and $W^{m} \subseteq W^{n - } \subseteq W \subseteq 2^{N}$. Therefore, $W^{n - }$ can be seen as either a restriction of *W* or as an extension of $W^{m}$. The aim of comparing these three indices is to try to explain how changes in the types of considered coalitions in the index influence the characteristic of the index.

By definition, all of the indices considered (*h*, *θ*, and *g*^*np*^) are non-negative and relative (i.e., range from 0 to 1).

### Theorem 2

For each simple game *v* and every non-null player $i \in N$, the following inequality holds: $\theta_{i} (v) \le g_{i}^{np} (v)$.

### *Proof*.

The demonstration immediately follows from the definition of the *g*^*np*^ index and Formula (1); i.e., $g_{i}^{np} (v) = \frac{{|W_{i}^{n - } |}}{{\sum\limits_{l \in N} {|W_{l}^{n - } |} }} \ge \frac{{|W_{i}^{n - } |}}{{\sum\limits_{l \in N} {|W_{l}^{n - } | + \frac{k}{2}|W^{n - } |} }} = \theta_{i} (v)$ for each non-null player $i \in N$. If we have *k* > 0 null players in a simple game, for each null player $i \in N$, $g_{i}^{np} (v) = 0 \le \frac{{|W^{n - } |}}{{2\sum\limits_{j \in N} {|W_{j}^{n - } | + k|W^{n - } |} }} = \theta_{i} (v)$. □

Let us consider some known properties of the power indices mentioned in Sect. 2. For every simple game *v* and each player $i \in N$, $g_{i}^{np} (v) \ge 0$ and $\theta_{i} (v) \ge 0$. Thus, the non-negativity property is satisfied by both indices: *θ* and *g*^*np*^*.* Moreover, the PHI *θ* satisfies the positivity property, which is stronger than non-negativity, i.e. $\theta_{i} (v) > 0$ for every $i \in N$, while *g*^*np*^ and *h* fail this property.

The symmetry property imposes that the power index does not depend on the labeling of the players. Thus, “symmetric” players should be assigned the same power in the game. The *h*, *θ*, and *g*^*np*^ indices satisfy the symmetry property.

The efficiency property requires that the players’ power values add up to 1 for all simple games. Like symmetry, the efficiency property is used in the axiomatic characterization of the *h*, *θ*, and *g*^*np*^ indices (see Holler and Packel 1982; Bertini et al. 2008; Álvarez-Mozos et al. 2015).

The null player property requires that the power index assigns zero power to a player with no influence on the worth of any coalition $S \in 2^{N} .$ The *g*^*np*^ index satisfies the null player postulate by definition. It is actually pretty obvious that *θ* does not satisfy the null player property: $v(N) = 1$ for every simple game, so it is immediately clear that every null player is part of at least one winning coalition and thus enjoys positive power according to *θ* (see Formulas (1), (2), and (3)). The *h* index satisfies the null player property (see Holler (1982), for example).

The power value assigned to player *i* by the PHI *θ* index depends on the number of null players in a game (see Formula (1)). So, the null player removable property is not satisfied by *θ*, but the *h* and *g*^*np*^ indices satisfy it by definition.

Even if the dominance property is not satisfied by the Holler index (1982), *θ* satisfies it (see Bertini et al. 2008; Bertini et al. 2013).

### Theorem 3

The *g*^*np*^ index satisfies the dominance property.

### *Proof.*

Consider weighted game *v* = $[q;w_{1} ,w_{2} ,\;...,w_{n} ]$, $n \ge 2$, and two non-null players $i,j \in N$ such that $i \ne j$ and $w_{i} \ge w_{j}$. Let us denote a set of all null player free winning coalitions containing both players *i* and *j* by $W_{i \cup j}^{n - }$ = $\{ S \in W^{n - } :i \in S \wedge j \in S\}$. Of course, $W_{i \cup j}^{n - }$ is non-empty, as players *i* and *j* are not null and $W_{i \cup j}^{n - }$ belongs to both $W_{i}^{n - }$ and $W_{j}^{n - }$. Next, $w_{i} \ge w_{j}$ implies that, for each non-empty coalition $S \in W_{j}^{n - } \backslash W_{i \cup j}^{n - }$, the following holds: $S\backslash \{ j\} \cup \{ i\} \in W_{i}^{n - }$. So, $\left| {W_i^{n - }} \right| \geqslant \left| {W_j^{n - }} \right|\,$. From this, we immediately have that $g_{i}^{np} (v) = \frac{{|W_{i}^{n - } |}}{{\sum\limits_{l \in N} {|W_{l}^{n - } |} }} \ge \frac{{|W_{j}^{n - } |}}{{\sum\limits_{l \in N} {|W_{l}^{n - } |} }} = g_{j}^{np} (v)$. □

Informally, the donation property states that a player who donates votes to another player (recipient) should not increase his voting power. Bertini et al. (2013) presented an example of a failure of the donation property for the PHI *θ* and Holler indices. The same example can be used to also show that the *g*^*np*^ index presents a failure of the donation property. Namely, let us consider two games *v*_1_ = [9; 6, 4, 1, 1, 1] and *v*_2_ = [9; 5, 5, 1, 1, 1]. Game *v*_2_ arises from *v*_1_ after the donation of one vote by Player 1 to Player 2. As game *v*_1_ is free of null players, both the *θ* and *g*^*np*^ indices give the same distribution of power. In particular, $\theta_{1} (v_{1} ) = g_{1}^{np} (v_{1} ) = \frac{9}{32}$ and $h_{1} (v_{1} ) = \frac{1}{3}$. After the donation from Player 1 to Player 2, the distribution of player weights change so that Players 3, 4, and 5 become null players. In game *v*_2_, Players 3, 4, and 5 (with one vote each) are not critical in any coalition. Moreover, in game *v*_2_, we have only one null player free winning coalition: {1, 2}, which is also a unique minimal winning coalition. Thus. $g_{1}^{np} (v_{2} ) = h_{1} (v_{2} ) = \frac{1}{2}$, and $\theta_{1} (v_{2} ) = \frac{2}{7}$. Therefore, Player 1 increases his/her power after the donation according to the *h*, *θ*, and *g*^*np*^ indices. So, all of these indices violate the donation property.

Felsenthal and Machover (1998, p. 232) also considered a stronger version of the donation property—the redistribution property (i.e., when more than one donor or one recipient could appear). The *h*, *θ*, and *g*^*np*^ indices present a failure of the donation property, so they will also present a failure of the redistribution property. Moreover, Felsenthal and Machover (1998, p. 253) considered the so-called *fattening property*. Informally, this property states that a player who puts on extra weight should not lose power. If we consider two weighted games *v*_1_ = [9; 5, 4, 1, 1, 1] and *v*_2_ = [9; 6, 4, 1, 1, 1], we can observe the failure of the fattening property for *h*, *θ*, and *g*^*np*^. Namely, $\theta_{1} (v_{1} ) = \frac{2}{7}$, $h_{1} (v_{1} ) = g_{1}^{np} (v_{1} ) = \frac{1}{2}$ and $g_{1}^{np} (v_{2} ) = \theta_{1} (v_{2} ) = \frac{9}{32}$, $h_{1} (v_{2} ) = \frac{1}{3}$. To paraphrase Felsenthal and Machover (1998)—A measure displaying the fattening paradox thereby violates Young’s (1985) strong monotonicity condition. So, the *h*, *θ*, and *g*^*np*^ indices violate the strong monotonicity property, which can be observe in the same example of games used to show the failure of the fattening postulate.

Bertini et al. (2013) provided an example that showed that *h* and *θ* violate the transfer property proposed by Dubey (1975). The same example can be used to show a failure of the transfer property for *g*^*np*^, since the considered games are free of null players.

Bertini et al. (2013) demonstrated that *h* and *θ* satisfy the bicameral meet property. Similarly, it could be shown that *g*^*np*^ also fulfills this property.

### Theorem 4.

The *g*^*np*^ index satisfies the bicameral meet property.

### *Proof.*

Let us consider three simple games $$v_{1} = (N_{1} ,v_{1} )$$, $$v_{2} = (N_{2} ,v_{2} )$$, $$v = (N,v)$$ such that $$N = N_{1} \cup N_{2}$$, $$N_{1} \cap N_{2} = \emptyset$$, $$W(v)$$$$= \{ S \in 2^{N} :S \cap N_{1} \in W(v_{1} )$$, and $$S \cap N_{2} \in W(v_{2} )\}$$. By the definitions of *N* and $$W(v)$$, it follows that the set of null player free winning coalitions in *N* is a Cartesian product of the null player free winning coalitions in the two separate games ($$v_{1}$$, $$v_{2}$$). So, the following equations hold for every $$i \in N_{1}$$ $$|W_{i}^{n - } (v)| = |W_{i}^{n - } (v_{1} )| \cdot |W^{n - } (v_{2} )|$$. Thus, for any non-null players $$i,j \in N_{1}$$, we have:

$$\frac{{g_{i}^{np} (v)}}{{g_{j}^{np} (v)}} = \frac{{|W_{i}^{n - } (v)|}}{{|W_{j}^{n - } (v)|}} = \frac{{|W_{i}^{n - } (v_{1} )| \cdot |W^{n - } (v_{2} )|}}{{|W_{j}^{n - } (v_{1} )| \cdot |W^{n - } (v_{2} )}} = \frac{{g_{i}^{np} (v_{1} )}}{{g_{j}^{np} (v_{1} )}}$$. □

Bertini and Stach (2015) showed that for any simple game $v \in S_{N}$, $\frac{1}{2n - 1} \le \theta_{i} (v) \le \frac{2}{n + 1}$ for any $i \in N$. The minimum PHI *θ* index a null player *i* can achieve in game $v \in S_{N}$ is shown in Theorem 5 and Corollary 3.

### Theorem 5

 If $v \in S_{N}$ and $1 \le k < n$, then the following inequality holds: $\theta_{i} (v) \ge \frac{1}{2n - k}$, where *i* is a null player and *k* is the number of null players in game *v*.

### *Proof.*

Let us fix an *n*-player simple game with *k* null players ($1 \le k < n$) such that the set of null player free winning coalitions is not empty, i.e. $W^{n - } \ne \emptyset$. Let us consider an arbitrary null player. Then from formula (1) we have

4 $$\theta_{i} (v) = \frac{{|W^{n - } |}}{{2\sum\limits_{j \in N} {|W_{j}^{n - } |} + k|W^{n - } |}}= \frac{1}{{2\sum\limits_{j \in N} {\frac{{|W_{j}^{n - } |}}{{|W^{n - } |}}} + k}}$$

Now we demonstrate that the minimal power that an arbitrary null player *i* can obtain in a simple game is equal to $\frac{1}{2n - k}$. The PHI index $\theta$ for null player *i* has a minimal value if the denominator of (4) attains a maximal value. The maximal value of denominator (4) is attain for maximal values of $\sum\limits_{j \in N} {\frac{{|W_{j}^{n - } |}}{{|W^{n - } |}}}$. Since the maximal value of $\frac{{|W_{j}^{n - } |}}{{|W^{n - } |}}$ is 1 for every $j \in N$, we see that the maximal value of $\sum\limits_{j \in N} {\frac{{|W_{j}^{n - } |}}{{|W^{n - } |}}}$ is $(n - k)$. Thus, from (4) we have:

5 $$ \theta_{i} (v) = \frac{1}{{2\sum\limits_{j \in N} {\frac{{|W_{j}^{n - } |}}{{|W^{n - } |}}} + k}} \ge \frac{1}{2(n - k) + k} = \frac{1}{2n - k} $$

Note that, for a given $n > 1$, where $1 \le k < n$, the quotient $\frac{1}{2n - k}$ attains the lowest value for *k* = 1. So, taking into account (5) we can formulate the following corollary.

### Corollary 3

The minimum PHI *θ* index a null player *i* can achieve in game $v \in S_{N}$ is equal to $\frac{1}{2n - 1}$, what happens when only one player is a null player in *v*.

### *Proof.*

The demonstration immediately follows from Theorem 5.

Table 3 summarizes the information of the three considered indices and the desirable properties reviewed in this paper. In Table 3, “yes” means that an index satisfies a particular property, and “no” means otherwise. Taking only the number of properties satisfied by the *g*^*np*^ index into account, we see that this index is closer to the Holler index than to the PHI *θ* index. The difference between these indices lies in the dominance property. Meanwhile, the *θ* and *g*^*np*^ indices differ in their null players, null player removable, and positivity properties.

**Table 3:** Power indices in comparison

Property	Power index		
	*h*	*θ*	*g*^*np*^
Bicameral meet	Yes	Yes	Yes
Dominance	No	Yes	Yes
Donation	No	No	No
Efficiency	Yes	Yes	Yes
Fattening	No	No	No
Null player	Yes	No	Yes
Null player removable	Yes	No	Yes
Positivity	No	Yes	No
Redistribution	No	No	No
Strong moNotonicity	No	No	No
Transfer	No	No	No
Range	[0, 1]	$[\frac{1}{2n - 1},\frac{2}{n + 1}]$	[0, 1]

## Conclusions

In this paper, we concentrate on two power indices: the PHI *θ* (Bertini et al. 2008), and the *g*^*np*^ index proposed by Álvarez-Mozos et al. (2015). Both indices can be seen as modifications of the PGI index (Holler 1982). The PGI index is based on the minimal winning coalitions, whereas *θ is* based on the winning coalitions and *g*^*np*^ indices on null player free winning coalitions.

The important contribution of this paper is the novel approach to calculate and express the PHI *θ* index. The new formula proposed in Sect. 3 for calculating the PHI *θ* uses only null player free winning coalitions. It should be note that the set of these coalitions unequivocally defines a simple game. In a transparent way, the new formula shows the power assigned to null and non-null players. The set of null player free winning coalitions has a non-greater cardinality than the set of all winning coalitions. Therefore, Formula (1) given in Sect. 3 can facilitate the calculations of PHI *θ* for a large number of players with the presence of null players in a simple game (see Sect. 4 in particular). This means that eliminating the null players from a game gives the advantage in reducing the space complexity of an algorithm that is used. Furthermore, if the representation of the weighted game is transformed into a minimal weighted representation in integers, then the null players are exactly those who have a weight equal to zero (see Freixas and Kurz (2014), for example), if there are some of them then it makes sense to apply the new formula proposed in this paper. An alternative easy way to identify null players is by means of classical indices, as the Banzhaf or Shapley-Shubik ones. Then, the null players are exactly ones who are assigned 0 as a payoff. Moreover, when a simple game is defined by the set of null player free winning coalitions, the calculation of the *θ* index by Formula (1) is immediate.

Moreover, we compare the *h*, *θ*, and *g*^*np*^ indices by taking some desirable properties for the power indices in simple games into account (see Sect. 5). In this way, we obtain a picture of the differences of the considered indices. Note that, for simple games without null players, *θ* and *g*^*np*^ are equal. In particular, we proved that the *g*^*np*^ index satisfies the dominance and bicameral meet properties (see Theorems 3 and 4).

One of the further developments is following the ideas of Freixas (2005a, 2005b, 2012, 2020) and Freixas and Pons (2021) and provide an extension of the PHI *θ* index for games with several levels of approval, for example. The next idea is a modification of *θ* to simple games with the known probability distribution over coalitions and then study the properties of the obtained index, see Freixas and Pons (2017), for example.

## Footnotes

1 The assumption of monotonicity is not needed for some of the theory of simple games, but as it seems to be a natural condition, we include it in the definition.
